# Toward Spatially Regulated Division of Protocells: Insights into the *E. coli* Min System from* in Vitro* Studies

**DOI:** 10.3390/life4040915

**Published:** 2014-12-11

**Authors:** Simon Kretschmer, Petra Schwille

**Affiliations:** Department of Cellular and Molecular Biophysics, Max Planck Institute of Biochemistry, Am Klopferspitz 18, Martinsried 82152, Germany; E-Mail: kretschmer@biochem.mpg.de

**Keywords:** protocell, bottom-up synthetic biology, cell division, Min proteins, membranes, self-organization, pattern formation

## Abstract

For reconstruction of controlled cell division in a minimal cell model, or protocell, a positioning mechanism that spatially regulates division is indispensable. In *Escherichia coli*, the Min proteins oscillate from pole to pole to determine the division site by inhibition of the primary divisome protein FtsZ anywhere but in the cell middle. Remarkably, when reconstituted under defined conditions *in vitro*, the Min proteins self-organize into spatiotemporal patterns in the presence of a lipid membrane and ATP. We review recent progress made in studying the Min system *in vitro*, particularly focusing on the effects of various physicochemical parameters and boundary conditions on pattern formation. Furthermore, we discuss implications and challenges for utilizing the Min system for division site placement in protocells.

## 1. Introduction

A defining aim of synthetic biology is the construction of a minimal cell that fulfills the most elementary requirements of life: self-maintenance, reproduction and potentially evolvability [1]. Toward this end, one can conceive of two complimentary approaches. In the top-down approach, the complexity of a natural cell is reduced through a series of iterative genetic deletions. Ideally, one eventually arrives at a minimal cell that lacks all non-essential genes under a given set of laboratory growth conditions [2]. For this category, the best-studied model organism so far is the bacterium *Mycoplasma genitalium* whose genome is already small, with about 500 genes, and thus constitutes a suitable starting point for further reduction [3,4].

In contrast, the bottom-up synthetic biology approach aims at (re-)constructing a functional cell from scratch using a minimal set of functional modules and parts under defined conditions [5]. In this framework, complexity is increased during the building process. The functional elements in such a bottom-up approach can be biological, purely chemical, or a combination of both [6]. A cell-like ensemble may form by either self-assembly of amphiphilic molecules into a compartment or by encapsulation of biomolecules into pre-formed liposomes [6,7,8,9]. The latter strategy has been termed the “reconstruction” approach, because it uses proteins and DNA from contemporary organisms (as in the top-down approach) but still leads to an increase in biological complexity [1]. While the top-down approach seeks to identify a minimal set of genes for life, the bottom-up strategy promises to elucidate the basic physicochemical mechanisms and general principles of cellular life. By building a life-like entity with known building blocks, the reconstruction approach can thus inform us how a living system could function in a very simple form [5].

Among other research interests in the bottom-up category, recent efforts have focused on the reconstruction of the minimal molecular machinery for cell division [10]. Ideally, such a machinery would be modular in the sense that it contains design principles which enable division of a variety of cell-like compartments. A minimal module to reconstitute spatiotemporally controlled division would have to comprise at least elements for generation of constriction force, as well as a mechanism for regulation of division in space and time. In order to reveal design principles for such a simplified system, the well-studied laboratory bacterium *Escherichia coli* constitutes a promising starting point. Even though *E. coli* did not arise at the beginning of evolution and is thus not an ideal model for investigating the origin of life, its internal organization might still reveal important clues regarding the essential mechanistic principles of division that might be used in protocell design.

Division of *E. coli* cells is enabled by the divisome, “the complete macromolecular machinery able to effect division in the living cell” [10]. The initial step of divisome assembly is assumed to be the formation of the so-called proto-ring composed of the presumably constriction force-generating tubulin-homolog FtsZ as well as FtsA and ZipA which anchor FtsZ to the membrane [10,11]. The proto-ring then serves as a scaffold for the assembly of other essential division proteins [12].

In order to localize the future division site to mid-cell, *E. coli* employs two independent mechanisms that negatively regulate proto-ring assembly at the poles: nucleoid occlusion and the MinCDE system [13,14]. Nucleoid occlusion inhibits Z-ring formation through the action of the protein SlmA [15]. The Min system is composed of the FtsZ inhibitor MinC as well as the membrane ATPase MinD and its activator MinE [16,17,18,19]. Remarkably, the Min proteins oscillate between the cell poles *in vivo* [20,21]. Furthermore, *in vitro* reconstitution of the Min dynamics could reproduce many of the key characteristics of the Min system making it a promising candidate for regulating division site placement in protocells (see Section 3 and Section 4).

## 2. The *E. coli* Min System

The Min system selects the middle of the cell for cytokinesis by inhibiting division at the cell poles in a spatiotemporal fashion. Accordingly, mutations in the *MinB* operon encoding MinC, MinD and MinE often result in a phenotype characterized by minicells which lack DNA and result from division at the poles [22]. In this section, we provide a brief overview of the biochemical properties of the Min proteins which have been reviewed in detail elsewhere [23,24,25].

Direct inhibition of Z-ring formation is conferred by MinC which is composed of independent N- and C-terminal domains [26]. The N-terminal domain interferes with FtsZ-FtsZ monomer interactions within an FtsZ polymer, while the C-terminal domain targets the lateral association of FtsZ protofilaments with additional roles in MinC dimerization and MinD interaction [16,25,26,27,28,29]. In order to localize this inhibitory activity to the cell poles and thus allow Z-ring formation exclusively at mid-cell, MinC oscillates together with MinD and MinE from cell pole to cell pole, creating a time-averaged concentration minimum of the inhibitor MinC in the cell middle [20,30].

MinD is a peripheral membrane-binding ATPase of the Walker A cytoskeletal ATPase (WACA) family [17,31]. Hydrolysis of ATP by MinD provides the energy for the spatiotemporal dynamics. Membrane binding is controlled by ATP-dependent dimerization of MinD and mediated by a membrane targeting sequence (MTS) in the form of a short C-terminal amphipathic helix [32,33,34,35]. Although the exact mechanism of how MinD interacts with the membrane is still unclear, it is well established that MinD membrane binding is cooperative [32,36].

MinE acts as an activator of MinD’s ATPase activity, which triggers detachment from the membrane [19,32]. It is composed of an N-terminal anti-MinCD domain able to counteract the inhibitory action of MinC and MinD as well as a C-terminal topological specificity domain responsible for regulating division spatially [37,38,39,40]. As the binding sites for both proteins overlap, MinE can displace MinC from membrane-bound MinD [32,35,41]. Remarkably, MinE can also interact with the membrane through an N-terminal nascent amphipathic helix functioning as an MTS [36,42,43]. An X-ray crystallographic study has implied that MinD-stabilized structural changes in MinE are key processes for driving the dynamics of the two proteins on the membrane [43]. In the absence of MinD, MinE exists in a cytoplasmic 6β-structure with the MTS buried in the core of the protein [43,44,45]. However, sensing of membrane-bound MinD induces a conformational change to a 4β-structure [43,46]. In this conformation, MinE can not only interact with MinD but can also bind the membrane after unmasking of MinE’s MTS [43]. By staying attached to the membrane, MinE can processively activate several MinD dimers on the membrane. Recently, it was also suggested that MinE self-assembly on the membrane plays a role for the Min system’s function [47].

These biochemical properties of MinD and MinE form the molecular basis for the intriguing spatiotemporal dynamics of the Min system. The oscillations of Min proteins in *E. coli* were first observed with fluorescent protein fusions of the Min proteins *in vivo* [20,21]. Initially, MinD cooperatively binds to the membrane and forms a growing cap at one cell pole while MinE forms a structure known as the E-ring at the rim of this polar zone [48,49]. After MinE-induced shrinking and disassembly of the polar zone, MinD and MinE diffuse through the cytoplasm to repeat this process at the other cell pole. MinC is not involved in generating these dynamics but acts as a passenger by following the oscillations of MinD and MinE [20,21]. These cycles of collective membrane association, dissociation and cytoplasmic diffusion take around 1–2 min [21,48]. As they are iteratively repeated during the *E. coli* growth and division cycle, a time-averaged concentration minimum of MinC is generated in the cell middle, thus selecting it for Z-ring assembly [20,30].

Besides positioning the divisome spatially, the Min system has also been shown to influence the timing of cell division [50]. Furthermore, it has been proposed that it is involved in chromosome segregation through binding of MinD to DNA [51].

## 3. Reconstitution of the Min System* in Vitro*

### 3.1. Self-Organization of Min Proteins into Surface Waves

**Figure 1:**
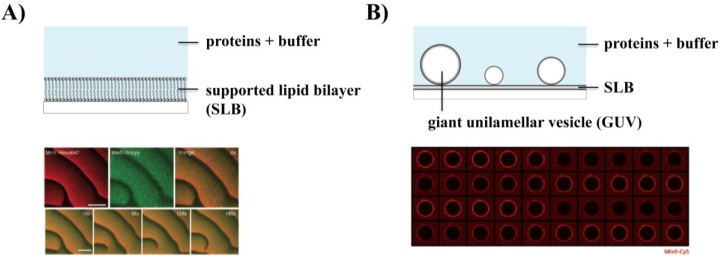

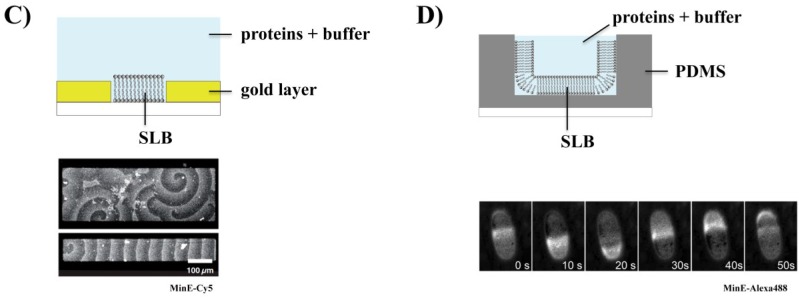
Reconstitution of Min protein dynamics under different constraints *in vitro*. (**A**) Self-organization into surface waves on a supported lipid bilayer (From reference [52]. Reprinted with permission from AAAS); (**B**) When reconstituted on free-standing membranes (GUVs), Min waves can be visualized as an on/off pattern in the equatorial plane (Reprinted with permission from [56], © 2013 Society for Applied Microbiology and John Wiley and Sons Ltd, Hoboken, NJ, USA); (**C**) Min waves propagate along the longest axis when confined in 2D (Reprinted with permission from [57]); (**D**) Pole-to-pole oscillations can be reconstituted in compartments with bacteria-like shape (Reprinted with permission from [58], © 2013 Wiley-VCH Verlag GmbH and Co., KGaA, Weinheim, Germany).

A fascinating hallmark of the Min system is that very rich and complex dynamic behavior emerges from the interactions of only a few components. When initially reconstituted on a supported lipid bilayer (SLB) mimicking the cytoplasmic membrane of *E. coli*, the Min proteins were found to self-organize into propagating planar surface waves (Figure 1A) [52]. These intriguing spatiotemporal patterns form by self-organization of MinD and MinE on the membrane with the necessary energy provided by ATP hydrolysis. The waves arise from an initially uniform distribution of ATP-bound MinD on the membrane by a reaction-diffusion mechanism, as MinE induces small local fluctuations of the MinE/MinD concentration ratio that get quickly amplified by positive feedback [23,52]. Remarkably, the Min patterns are strongly reminiscent of other self-organization phenomena with examples ranging from aggregation of the slime mould *Dictyostelium discoideum* to chemical self-organization in the Belousov-Zhabotinsky reaction or the catalytic oxidation of carbon monoxide on platinum surfaces [23,53,54,55]. In turn, the Min system has become a paradigm for protein self-organization.

Interestingly, the dynamic patterns formed by MinD and MinE on the membrane reproduced many of the *in vivo* characteristics of the Min oscillations, in spite of their *ca.* 10 times larger spatial scale *in vitro*. Firstly, the temporal period is roughly similar to the *in vivo* dynamics with around 1–2 min and sensitive to the MinE/MinD concentration ratio [52,59]. Furthermore, MinE accumulates as a sharp band at the rear of the wave, reminiscent of the E-ring *in vivo* [48,49,52]. A follow-up study showed that MinC is not required for generating the dynamic cycling of the Min proteins on the membrane but acts as a passenger [10,60]. Furthermore, the order of events during wave propagation was dissected [60]. It was found that MinE accumulates at the rear of the wave prior to MinC and MinD detachment. Additionally, FRET experiments ruled out the possibility of MinE monomers binding to membrane-attached MinD before dimerizing to form the E-ring, while FRAP argued against cooperative MinE binding. This implied that MinE is incorporated into the waves as a dimer and accumulates because of wave propagation [60]. Strikingly, single-particle tracking of labeled Min proteins on the membrane showed that, besides lower diffusivities of MinD and MinE at the rear of the wave, MinE displayed a longer residence time on the membrane than MinD. This argued for a mechanism where MinE accumulates because of persistent binding. In this mechanistic view, MinE rapidly rebinds the membrane after activation of a membrane-bound MinD dimer. This gives rise to positive feedback during MinD detachment which is likely to be a major factor for symmetry breaking of the homogenous state as well as wave propagation [60]. Furthermore, by analyzing a MinE mutant deficient in membrane binding that self-organizes into asynchronous patterns with MinD, it was suggested that MinE membrane binding contributes to the robustness of pattern formation [60].

As stated above, the *in vitro* Min patterns share key similarities with the oscillations *in vivo*. Yet, the successful reconstitution on SLBs also raised several important questions about observed differences, as well as the role of physicochemical parameters and boundary conditions.

### 3.2. Effect of Physicochemical Parameters on Min Patterns

As mentioned above, a striking difference between the oscillations in cells and the waves on SLBs is that the wavelength *in vitro* is about ten times higher than *in vivo*. It was suggested that this could be due to either higher reaction rates or slower protein diffusion on the membrane inside an *E. coli* cell [23]. To test if diffusion is the defining cause for the difference in the wavelength, Min patterns were recently reconstituted on free-standing membranes [56]. In this study, giant unilamellar vesicles (GUVs) were used which, due to their size, provide a quasi-planar surface. Furthermore, unlike SLBs, they are not subject to unwanted effects from a solid support, as both sides of the bilayer are exposed to solution. As the diffusion of lipids and other molecules is several times faster on GUVs than on SLBs, they constitute an ideal model membrane to quantitatively compare the effects of diffusivities on membrane protein dynamics [61]. By incubating Min proteins with GUVs, circular and spiral waves were observed on the outer surface of free-standing membranes [56]. When imaging the equatorial plane of the GUVs, propagation of the Min waves could be visualized as a characteristic on/off pattern of the fluorescence intensity (Figure 1B). Remarkably, it was demonstrated that while the temporal period was similar between GUVs and SLBs, the wavelength and velocity were increased by about a factor of 3 on free-standing membranes, consistent with a higher diffusion coefficient of MinD on GUVs compared to SLBs as determined by FRAP [56]. This suggested that Min protein diffusion on the membrane is indeed a determinant of the wavelength and could potentially account for the difference in length scales between the *in vivo* and *in vitro* systems [56]. Nevertheless, it would be interesting to analyze the dependence of the wave parameters on different reaction rates to determine their contribution to the larger length scale *in vitro*.

Besides protein diffusion on the membrane, other physicochemical factors shown to influence Min dynamics *in vitro* include flow as well as lipid composition and salt concentration [62,63]. Experiments on the influence of flow suggested that Min proteins do not self-organize into regular waves but instead form a variety of other interconverting patterns when flow is externally applied [62]. By testing various lipid compositions, it was found that the anionic charge density of the membrane affects Min waves and that the lipid cardiolipin is not required for Min dynamics [63]. In combination with results from experiments on the influence of varying ionic strength, it was determined that MinD, and to a greater extent MinE, preferentially bind to anionic phospholipids and that an increase in anionic phospholipids or a decrease in salt concentration results in a lower wavelength and velocity. At very high densities of negative membrane charge or low ionic strength, MinE can even bind to the membrane independent of MinD [63].

### 3.3. Effect of Geometry on Min Patterns: From Waves to Oscillations

Another critical factor regulating Min protein self-organization was proposed to be the membrane topology and geometry. In a first study, it was investigated how two-dimensional geometrical cues and boundaries affect the Min patterns by reconstituting Min proteins on photolithographically micropatterned membranes with well-defined geometry [57]. Strikingly, when reducing the size of the membrane patches to a width similar to the Min patterns, the waves sense the membrane geometry and select the longest axis as the preferential direction of propagation (Figure 1C). This demonstrated that two-dimensional constraints are sufficient to determine the wave directionality, with no need for three-dimensional confinement. It was also shown that Min waves are capable of coupling laterally across small pre-defined gaps in the membrane. Interestingly, if gaps are made sufficiently large or the diffusive exchange of particles is limited via crowding agents in solution, the waves do not display coupling [57].

Besides probing the influence of the surrounding two-dimensional geometry on the patterns, another long-standing goal to bridge the *in vivo* and *in vitro* observations has been the reconstitution of Min oscillations under biomimetic conditions. Remarkably, pole-to-pole oscillations could indeed be reconstituted by incubating Min proteins in microfabricated picoliter-sized polydimethylsiloxane (PDMS) compartments mimicking the shape of *E. coli* cells. (Figure 1D) [58]. In these experiments, the dimensions of the chambers were adjusted to the roughly ten times bigger length-scale of the *in vitro* system. Apart from oscillations, characteristic patterns formed in spherical and filamentous cells could be reproduced in compartments mimicking these shapes [48,49,58,64,65]. The reconstitution of Min oscillations *in vitro* proved that the size and geometry of cells indeed modulates Min protein self-organization and corroborated that pattern formation *in vivo* and *in vitro* is governed by the same mechanism. Intriguingly, closing the membrane in three dimensions is not required in order to establish periodic oscillations from surface waves, but the only prerequisite is the limitation of volume, established by an air interface at the top of a membrane-clad compartment.

Furthermore, it was recently demonstrated that Min patterns can be aligned by topological cues. Using PDMS substrates with micron-sized grooves in the shape of concentric rings, a globally predefined orientation of the Min waves was achieved with alignment taking place early during pattern formation [66]. This established surface topology as another promising regulatory element of Min patterns *in vitro*.

### 3.4. Co-Reconstitution of the Min System with FtsZ

The *in vitro* reconstitution of force generation by FtsZ has been intensively studied and is described in other reviews, e.g., by Rivas *et al.* [67].

Importantly, it has been shown that FtsZ can form Z-rings inside tubular multilamellar liposomes and generate indentations even in the absence of its native membrane anchors FtsA and ZipA, when FtsZ is fused to a membrane targeting sequence [11]. A follow-up study showed that Z-rings can be observed when reconstituting the gain-of-function mutant FtsA* together with FtsZ, GTP and ATP in unilamellar liposomes [68]. Under these conditions, it was suggested that liposome constriction and in few cases division occurs [68]. Furthermore, a recent study demonstrated that, besides tethering FtsZ to the membrane, FtsA also promotes treadmilling of FtsZ, leading to self-organization into spiraling rings and other dynamic patterns on an SLB [69]. It appears that FtsA contributes to destabilization of FtsZ filaments, potentially by inferring topological strain when attaching them to the membrane, thus establishing the required negative feedback [70].

Regarding the co-reconstitution of FtsZ with the MinCDE system *in vitro*, it is crucial to understand the mechanism by which MinC inhibits FtsZ polymerization. By reconstituting a dynamic FtsZ network on SLBs and probing the effect of MinC by a range of optical techniques, it was shown that MinC exploits the turnover dynamics inherent to FtsZ filaments for its inhibitory activity [71]. This is consistent with the previous finding that FtsZ’s GTPase activity is required for inhibition by MinC [16]. By blocking stochastically generated binding sites on FtsZ filaments, MinC effectively reduces the attachment rate of fresh FtsZ monomers and increases the detachment rate from filaments [71]. Furthermore, the reconstitution of the MinCDE system with FtsZ-MTS was achieved on SLBs. This demonstrated that localized inhibition of FtsZ can lower the concentration required for FtsZ filament disassembly [71]. It would now be interesting to quantitatively characterize localization and disassembly of FtsZ by multiple depolymerization factors such as MinC and FtsA.

Very recently, the reconstitution of a Min protein gradient, which directs the localization of FtsZ in a biomimetic compartment was achieved [72]. A time-averaged non-homogeneous MinD distribution with clear minimum in the middle of the compartment emerging from pole-to-pole oscillations was reconstituted under minimal conditions in a compartment with cell-like shape. In co-reconstitution experiments, such a gradient was also found for MinC. Remarkably, the gradient formed by the MinCDE system *in vitro* acts as a spatial cue for polymerization of FtsZ-MTS whose assembly is directed to the middle of the compartment. At this region, where the time-averaged concentration of MinC is lowest, FtsZ-MTS assembles into bundles which align perpendicular to the long axis, consistent with a previous study in which FtsZ-MTS was found to preferentially align along negatively curved supported membranes [72,73]. Intriguingly, it was also demonstrated that gradient formation depends on the geometry of the compartment. For example, it was shown that pole-to-pole oscillations of Min proteins are stable when the compartment length is increased by a factor of around two, consistent with requirements for a growing cell. However, at higher lengths, double oscillations and eventually even higher order oscillation modes occur. In the case of double oscillations, FtsZ-MTS assembly is triggered at two regions along the length of the compartment. This suggested a mechanism with which a transiently filamentous cell may be able to regain normal cell length through localized cytokinesis [72].

## 4. Conclusions and Outlook

In this short review, we have summarized the most recent results on the *in vitro* reconstitution of the Min system. After the initial reconstitution of Min waves on SLBs, several open questions have been answered. This has significantly bridged the gap between the *in vitro* and *in vivo* observations.

Regarding the roughly 10-times bigger length scale of the *in vitro* patterns on SLBs, experimental evidence suggested that this increase could be at least partially due to faster protein diffusion on the membrane *in vitro* [56]. It was also shown that MinD and MinE preferentially bind to anionic lipids with a higher preference attributed to MinE [63]. Furthermore, the role of the surrounding geometry has been clarified as Min patterns were found to respond to boundaries and select the longest axis for propagation [57]. The reconstitution of pole-to-pole oscillations in cell-like chambers constituted another important step toward mimicking the *in vivo* behavior of the Min system in an artificial context [58]. Additionally, the co-reconstitution of the Min system with FtsZ showed that the Min system is indeed sufficient to regulate FtsZ polymerization spatially *in vitro* [71]. Finally, the localization of FtsZ to the middle of a cell-like compartment by a MinC gradient demonstrated that the Min system is capable of directing the localization and assembly of downstream proteins to specific regions *in vitro* [72].

The obvious next step toward reconstituting spatially controlled division of a protocell is the co-reconstitution of the Min system with a contractile machinery based on FtsZ inside a deformable cell-like compartment. Such an ensemble would have to fulfill several important criteria. First, the proteins would need to be encapsulated into a liposome or other soft compartment with similar transformability. Besides the three Min proteins and FtsZ, membrane anchors for the latter are necessary and could be realized either by an FtsZ-MTS fusion or by adding the natural anchoring proteins FtsA and ZipA. Second, the vesicle should be deformable to adapt a rod-like shape, for the Min proteins to select the correct propagation axis and enable pole-to-pole oscillations. The vesicle should also include anionic lipids and be tolerable of physiological salt concentrations as well as crowding agents to replicate the conditions inside a living cell [56].

Toward the goal of encapsulation, one possible route is provided by the droplet transfer method with which protein-containing water-in-oil droplets can be converted into vesicles [74,75]. Another promising approach is microfluidic jetting. In this method, liposomes are simultaneously generated and loaded with molecular cargo through a pulsed microfluidic jet which deforms a planar membrane into a vesicle [76]. However, the confinement of Min proteins inside a compartment may limit the flexibility of establishing correct concentrations, compared to the systems reconstituted *in vitro* so far. As concentrations affect binding and unbinding rates, further challenges arise [56]. Thus, in order to reconstitute the dynamics of the Min system and FtsZ inside a vesicle, precise readjustment of concentrations and other parameters will be necessary.

Additionally, a more detailed understanding of the Min dynamics’ underlying mechanism could not only facilitate its use as a positioning system in protocells, but potentially guide the *de novo* design of an even more simplistic machinery for the placement of spatial cues. Toward this end, a systematic analysis of how exactly the biochemical properties of the Min system contribute to self-organization could identify important parameter values, such as rates of binding or enzymatic turnover.

Lastly, the emergence of complex patterns through the self-organization of a relatively small number of components, which interact in a non-linear fashion, has been established as a recurring feature of living systems [53]. As a functional biomimetic system most likely relies on such properties, further study of self-organization in the case of the Min system could elucidate design principles of fundamental relevance for the bottom-up reconstitution of biological modules.
